# Genetic variation in the estrogen metabolic pathway and mammographic density as an intermediate phenotype of breast cancer

**DOI:** 10.1186/bcr2488

**Published:** 2010-03-09

**Authors:** Jingmei Li, Louise Eriksson, Keith Humphreys, Kamila Czene, Jianjun Liu, Rulla M Tamimi, Sara Lindström, David J Hunter, Celine M Vachon, Fergus J Couch, Christopher G Scott, Pagona Lagiou, Per Hall

**Affiliations:** 1 Karolinska Institutet, Department of Medical Epidemiology and Biostatistics, Box 281, 171 77 Stockholm, Sweden; 2 Human Genetics, Genome Institute of Singapore, 60 Biopolis Street, Singapore 138672, Singapore; 3 Channing Laboratory, Brigham and Women's Hospital, 181 Longwood Avenue, Boston, MA 02115, USA; 4 Department of Epidemiology, Harvard School of Public Health, 677 Huntington Avenue, Boston, MA 02115, USA; 5 Program in Genetic and Molecular Epidemiology, Department of Epidemiology, Harvard School of Public Health, 677 Huntington Avenue, Boston, MA 02115, USA; 6 Department of Health Sciences Research, Mayo Clinic College of Medicine, 200 First Street SW, Rochester, MN 55905, USA; 7 Department of Laboratory Medicine and Pathology, Mayo Clinic College of Medicine, 200 First Street SW, Rochester, MN 55905, USA; 8 Department of Hygiene, Epidemiology and Medical Statistics, University of Athens Medical School, 75 Mikras Asias Str, Goudi, Athens 115 27, Greece

## Abstract

**Introduction:**

Several studies have examined the effect of genetic variants in genes involved in the estrogen metabolic pathway on mammographic density, but the number of loci studied and the sample sizes evaluated have been small and pathways have not been evaluated comprehensively. In this study, we evaluate the association between mammographic density and genetic variants of the estrogen metabolic pathway.

**Methods:**

A total of 239 SNPs in 34 estrogen metabolic genes were studied in 1,731 Swedish women who participated in a breast cancer case-control study, of which 891 were cases and 840 were controls. Film mammograms of the medio-lateral oblique view were digitalized and the software Cumulus was used for computer-assisted semi-automated thresholding of mammographic density. Generalized linear models controlling for possible confounders were used to evaluate the effects of SNPs on mammographic density. Results found to be nominally significant were examined in two independent populations. The admixture maximum likelihood-based global test was performed to evaluate the cumulative effect from multiple SNPs within the whole metabolic pathway and three subpathways for androgen synthesis, androgen-to-estrogen conversion and estrogen removal.

**Results:**

Genetic variants of genes involved in estrogen metabolism exhibited no appreciable effect on mammographic density. None of the nominally significant findings were validated. In addition, global analyses on the overall estrogen metabolic pathway and its subpathways did not yield statistically significant results.

**Conclusions:**

Overall, there is no conclusive evidence that genetic variants in genes involved in the estrogen metabolic pathway are associated with mammographic density in postmenopausal women.

## Introduction

Mammographic breast density is one of the strongest risk factors for breast cancer. Several studies have shown that women with extensive dense tissue are at two to six times higher risk of developing the disease than women of similar age with lower mammographic density [1,2]. A strong genetic basis has been suggested for mammographic density [3]. Twin studies have estimated the heritability of this trait to be between 60 and 67% [4]. Evidence for a genetic influence also comes from other studies on family history, familial aggregation and segregation analyses [5,6].

Mammographic density is strongly correlated with hormone exposure profiles of women [7]. Several hormonal risk factors for breast cancer have been found to influence mammographic density in a similar fashion to their respective associations with risk for the disease [8]. For example, a strong inverse relationship has been observed between parity on mammographic density [9]. In addition, hormone replacement therapy (HRT) users and women who have a late first-born child or late menopause have higher breast densities on average [9]. In view of evidence suggesting an association between mammographic density and hormone-related factors, and the fact that estrogen is a strong risk factor for postmenopausal breast cancer, efforts have been made to identify underlying genetic determinants of mammographic density within pathways related to steroid hormone biosynthesis and metabolism [10-13]. Such endeavors assume mammographic density to be an intermediate phenotype for breast cancer. Several genes involved in hormone-related pathways - such as HSD3B1 [5,14], COMT [11,14] and ESR1 [15] - have been suggested to be associated with mammographic breast density. Findings are inconsistent, however, and only few candidate genes have been studied at a time.

We recently reported the results of a study evaluating a total of 239 SNPs in 34 estrogen metabolic genes in 1,596 breast cancer cases and 1,730 population controls from Sweden, of which the outcome variable was breast cancer (Low *et al*., manuscript submitted). No significant SNP association was evident after correction for multiple testing, but pathway-based global tests revealed significant association evidence for the overall estrogen metabolic pathway (*P *= 0.034) and, in particular, the androgen-to-estrogen conversion subpathway (*P *= 0.007). In the present study, we comprehensively examine genetic variation in the estrogen metabolic pathway with mammographic density. The number of SNPs and genes studied provides the most extensive coverage to date with respect to studying mammographic breast density.

## Materials and methods

### Study subjects

The subjects included in the current study are drawn from a population-based case-control study of postmenopausal breast cancer in women born in Sweden aged 50 to 74 years at the time of enrollment, which was between 1 October 1993 and 31 March 1995. Controls were randomly selected from the Swedish Total Population Register and were frequency matched to the expected age distribution of the cases. Details on data collection and subjects have been described previously [16]. The final study group with both mammographic density and genotype data included 891 breast cancer cases and 840 controls. Although all women were postmenopausal at the time of recruitment to the parent study, a subset of the women (43/1,731) was premenopausal in reference to the date of mammogram.

Approval of the study was given by the ethical review board at the Karolinska Institutet (Stockholm, Sweden) and six other ethical review boards in the respective regions in which the subjects were based, and informed consent was obtained from each participant.

Validation of SNPs with significant associations was performed using mammographic density data from two other studies.

### Mammographic density data

The process of collecting mammographic density data in this study has been described previously [17]. Film mammograms of the medio-lateral oblique view were digitized using an Array 2905HD Laser Film Digitizer (Array Corporation, Tokyo, Japan), which covers a range of 0 to 4.7 optical density. For controls, the breast side was randomized. For cases, the side contralateral to the tumor was used. The density resolution was set at 12-bit spatial resolution. The Cumulus software used for the computer-assisted measurement was developed at the University of Toronto [18]. For each image, a trained observer (LE) set the appropriate gray-scale threshold levels defining the edge of the breast and distinguishing dense from nondense tissue. The software calculated the total number of pixels within the entire region of interest and within the region identified as dense. These values were used to calculate the percentage of the breast area that is dense. A random 10% of the images were included as replicates to assess the intra-observer reliability, which was high with a Spearman rank correlation coefficient of 0.95.

### Gene and SNP selection

The process of gene and SNP selection has been described in detail by Low *et al*. (manuscript submitted). A total of 1,007 SNPs were selected from 35 genes and their 30 kb flanking sequences that code the enzymes involved in estradiol or estrone metabolism and are expressed in the breast. These SNPs were genotyped in 92 Swedish control samples to assess linkage disequilibrium patterns, to select tagging SNPs (tagSNPs) and to evaluate their coverage.

Haplotypes were reconstructed using the partition-ligation-expectation-maximization algorithm [19] implemented in the *tagSNPs *program [20]. A subset of tagSNPs were selected based on the *R*^2 ^coefficient, which quantifies how well the tagSNP haplotypes predict the genotype or the number of copies of haplotypes an individual carries. The performance of tagSNPs in capturing unobserved SNPs within the genes was evaluated using a SNP-dropping analysis. In brief, each of the genotyped SNPs was dropped in turn and then tagSNPs were selected from the remaining SNPs so that their haplotypes predicted the remaining SNPs with an *R*^2 ^value of 0.85. In total, 312 tagSNPs from the 35 genes were selected for genotyping.

**Figure 1:**
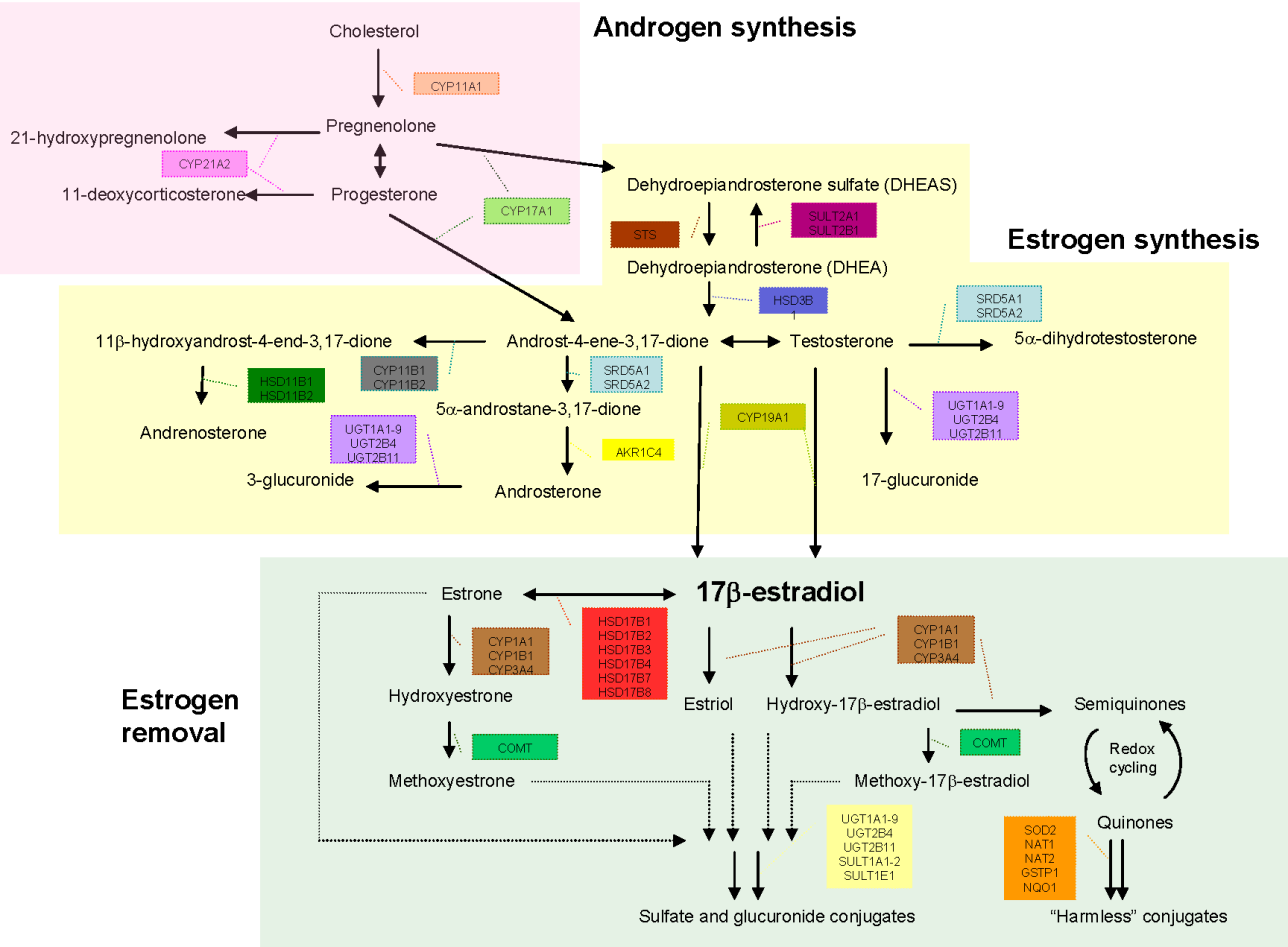
**Subdivision of the estrogen metabolic pathway**. The 34 metabolic genes analyzed in the present study are involved in different steps of the estrogen metabolism. The genes are divided into the three groups involved in androgen synthesis, estrogen synthesis and estrogen removal for subpathway-based association analysis.

Figure 1 delineates the processes and genes involved in the androgen synthesis, androgen-to-estrogen conversion and estrogen removal subpathways. The lists of SNPs corresponding to each subpathway are summarized in Tables S1 to S3 in Additional file 1.

### DNA extraction and genotyping

DNA was extracted from 4 ml whole blood using the QIAamp DNA Blood Maxi Kit (Qiagen, Hilden, Germany) according to the manufacturer's instructions and nonmalignant cells in paraffin-embedded tissue using a standard phenol/chloroform/isoamyl alcohol protocol. Genotyping was performed using the primer extension-based assay from Sequenom (San Diego, CA, USA) according to the manufacturers' instructions. DNA samples were randomly assigned to the plates carrying positive and negative controls, and all genotyping results were generated and checked by laboratory staff unaware of the case-control status. SNPs with a call rate <85%, minor allele frequency <1% or out of Hardy-Weinberg equilibrium (*P *< 0.05/312) were excluded from further analysis. The genotype concordance was >99%, suggesting high genotyping accuracy. Overall, 239 tagSNPs from the 34 genes were successfully genotyped and used in statistical analysis.

### Statistical analysis

Linear regression models were fitted, treating percentage density as an outcome. Models were adjusted for age, body mass index, menopausal status and HRT. Age was coded as 0, 1 and 2 for women <50 years, between 50 and 60 years, and >60 years of age, respectively. The body mass index was treated as a continuous variable. Menopausal status was determined from the time difference between the date of menopause and the date on which the mammogram was taken. HRT was considered a categorical variable made up of three groups: never users, past users and current users. The mammographic density measurements were transformed by the power of 0.3, yielding an approximately normal distribution. The genotypes were coded 0, 1 and 2 and treated as continuous variables.

A likelihood ratio test was performed for each SNP. Normal quantile-quantile plots were used to examine the distributions of the -log_10_-transformed *P *values. To assess whether the SNPs associated with breast cancer risk are the same SNPs as those associated with mammographic density, we used the Spearman's rank correlation test, evaluating the relationship between odds ratios corresponding to SNP effects on breast cancer risk and the regression coefficients of SNP effects on percentage density. The admixture maximum likelihood-based global test [21] was performed to evaluate the cumulative effect on mammographic density from multiple SNPs within the whole metabolic pathway and three subpathways for androgen synthesis, androgen-to-estrogen conversion and estrogen removal. Affection status for the admixture maximum likelihood analysis was defined by taking the lowest quantile of all percentage density measurements as controls and the highest quantile as cases. *P *values of the admixture maximum likelihood test were obtained via 5,000 permutations. Software R (v2.8.0) [22] and admixture maximum likelihood [21] were used for data management, quality control and statistical analyses.

### Validation of significantly associated SNPs

SNP associations with mammographic density were validated in 1,590 women genotyped with the Illumina HumanHap500 as part of the Cancer Genetic Markers of Susceptibility Project (CGEMS) [23]. The CGEMS project is a National Cancer Institute initiative to conduct genome-wide association studies to identify genes involved in breast cancer and prostate cancer. The initial CGEMS breast cancer scan was designed and funded to study the main effect of SNP variants on breast cancer risk in postmenopausal women, and has been completed [24]. Briefly, the first stage of the project involved a whole genome scan of 1,145 invasive postmenopausal breast cancer cases and 1,142 matched controls from the Nurses' Health Study nested case-control study [24]. The Nurses' Health Study was initiated in 1976, when 121,700 US registered nurses aged 30 to 55 returned an initial questionnaire [25]. During 1989 and 1990, blood samples were collected from 32,826 women [26]. For 1,590 of these women - of which 806 were breast cancer cases and 784 were healthy controls - we also had mammographic density measurements.

We collected mammograms as close as possible to the date of blood collection (1989 to 1990). To assess mammographic density, the craniocaudal (CC) views of both breasts were digitized at 261 μm/pixel with a Lumysis 85 laser film scanner, which covers a range of 0 to 4.0 optical density. The software for computer-assisted thresholding was developed at the University of Toronto [18]. We used the average percentage density of both breasts for this analysis. This collection has been described in detail in a previous publication [27]. SNPs not available on the Illumina HumanHap550 panel were imputed using MACH [28] based on HapMap Phase II (release 21a). For the analysis of imputed data, the ProbABEL package from the ABEL set of programs was used [29]. Percentage density was transformed by the power of 0.3 to be consistent with the parent study. This study was approved by the Committee on the Use of Human Subjects in Research at Brigham and Women's Hospital.

The second validation population consisted of a set of controls from an ongoing breast cancer case-control study at the Mayo Clinic. Briefly, the Mayo Clinic Breast Cancer Study is an Institutional Review Board-approved, clinic-based, case-control study initiated in February 2001 at Mayo Clinic, Rochester, MN, USA. The study design has been presented previously [30,31]. Clinic attendance formed the sampling frame for Mayo Clinic cases and controls. Consecutive cases were women aged 18 years or over with histologically confirmed primary invasive breast carcinoma and recruited within 6 months of the date of diagnosis. Cases lived in the six-state region that defines Mayo Clinic's primary service population (Minnesota, Iowa, Wisconsin, Illinois, North Dakota, and South Dakota). Controls without prior history of cancer (other than nonmelanoma skin cancer) were frequency matched on age (5-year age category), race and six-state region of residence to cases. Controls were recruited from the outpatient practice of the Divisions of General Internal Medicine and Primary Care Internal Medicine at Mayo Clinic, where they were seen for routine medical examinations.

The present analysis genotyped Caucasian controls (99% of study participants) enrolled through September 2007, who had mammograms available, representing 995 total controls (76% of total possible controls), of which 783 were postmenopausal. Screening mammograms were ascertained close to the enrollment date and the left CC view was digitized on an Array 2905HD Laser Film Digitizer, which covers a range of 0 to 4.7 optical density. Percentage mammographic density was estimated by an expert reader [32] on the left CC view, using the same Cumulus software described above [33]. Genotyping was carried out using TaqMan (Applied Biosystems, Foster City, CA) according to the manufacturer's instructions, using 10 to 20 ng DNA. Primers and probes were Assay-by Design (Applied Biosystems). Following PCR amplification, end reactions are read on the ABI Prism 7900 ht using Sequence Detection Software (Applied Biosystems). SNP associations were examined only in the 783 postmenopausal controls, to be comparable with the two other populations. The percentage density was transformed by the power of 0.3 to be consistent with the parent study.

## Results

Our dataset consisted of 1,731 postmenopausal women, of which 981 were breast cancer cases and 840 were controls (Table 1). Cases and controls differed significantly in age at first birth (*P *= 0.0126), parity (*P *< 0.0001), family history of breast cancer (*P *= 0.0002) and percentage density (*P *= 0.0017). Cases were found to have higher percentage density (mean ± standard deviation: 16.7 ± 14.3) than controls (14.6 ± 14.0). No significant difference was found for age, height, weight, body mass index, age at menarche, age at menopause or HRT usage.

**Table 1:** Selected characteristics of subjects

	Breast cancer cases (n = 891)		Breast cancer controls (n = 840)		
			
	Mean	SD	Mean	SD	*P *value
Age (years)	63.0	6.3	63.0	6.3	0.9045
Height (cm)	164.1	5.7	163.6	5.5	0.0766
Weight (kg)	68.9	110	68.8	11.6	0.8153
Body mass index	25.6	3.9	25.6	4.1	0.8420
Age at menarche (years)	13.6	1.4	13.6	1.5	0.6090
Age at first birth (years)	25.4	50	24.8	4.7	0.0126
Parity	1.9	1.2	2.2	1.3	0.0000
Age at menopause (years)	50.3	3.6	50.1	3.9	0.1223
HRT (% ever use)	0.53		0.50		0.2523
Family history (%)	0.15		0.09		0.0002
Percent density	16.7	14.3	14.6	14.0	0.0017

Means and standard deviations (SD) are given for continuous measures, proportions for other variables. *P *values based on the Welch *tt*est for independent samples. HRT, hormone replacement therapy.

**Figure 2:**
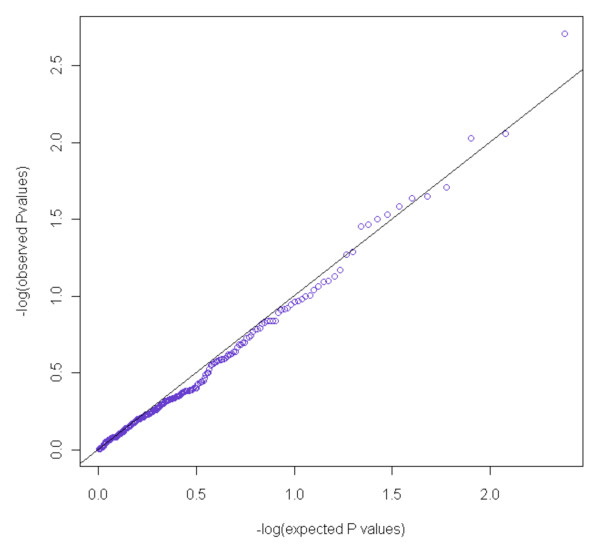
**No association between SNPs and percentage density**. -log_10 _quantile-quantile *P *value plots from single-SNP trend tests of 239 SNPs in the estrogen metabolism pathway.

Table S4 in Additional file 2 shows a list of 34 genes involved in the estrogen metabolic pathway and the corresponding number of SNPs examined for each gene. References are given for genes that have been examined in other studies for an association with mammographic density. Of the 239 SNPs analyzed, 11 SNPs were found to be significant at the 5% level (Table 2) - of which the smallest *P *value was 0.0019. Among six tagSNPs selected for the gene CYP11A1, five were found to be significant in the same direction. The associations in the single SNP analysis were moderate and would not survive correction for multiple SNP testing. In addition, the single-SNP *P *values showed no clear deviation from the null distribution, representing no association between SNPs and percentage density (Figure 2; see also Tables S1 to S3 in Additional file 1). None of the SNPs found to be nominally significant in our dataset were found to be significant in the CGEMS validation set (see Table S5 in Additional file 3). A second, independent validation carried out on the most significantly associated SNP (rs11638442) located within the CYP11A1 gene in 783 postmenopausal women with mammograms in the Mayo Clinic Breast Cancer Study yielded a *P *value of 0.88 (regression coefficient = -0.000507, 95% confidence interval = -0.07251 to 0.06237).

**Table 2:** Significant SNPs in the estrogen metabolic pathway, corresponding regression coefficients and *P *values

SNP	Gene	Minor allele	MAF	*n*	Coefficient	SE	*P *value
rs11638442	CYP11A1	C	0.35	1,677	0.0557	0.0212	0.0088
rs16968478	CYP11A1	G	0.17	1,703	0.0575	0.0263	0.0293
rs2279357	CYP11A1	A	0.20	1,699	0.0511	0.0229	0.0260
rs2959003	CYP11A1	A	0.28	1,669	0.0582	0.0224	0.0094
rs2959008	CYP11A1	A	0.30	1,703	0.0475	0.0221	0.0315
rs2066485	HSD17B3	G	0.14	1,703	0.0668	0.0293	0.0230
rs7039978	HSD17B3	A	0.50	1,694	--0.0632	0.0203	0.0019
rs1469908	NQO1	C	0.37	1,695	--0.0472	0.0206	0.0223
rs17268974	STS	A	0.22	1,605	0.0503	0.0238	0.0349
rs2270112	STS	C	0.34	1,686	--0.0485	0.0208	0.0197
rs707762	STS	A	0.40	1,687	0.0435	0.0205	0.0340

*P *values from a one-degree-of-freedom likelihood ratio test. MAF, minor allele frequency; SE, standard error.

Since the estrogen metabolic SNPs examined have previously been associated with breast cancer risk, we estimated the correlation between regression coefficients of SNP effects on mammographic density and the odds ratios of SNP effects on breast cancer risk, in order to assess whether the SNPs act through mammographic density as an intermediate phenotype for breast cancer. No significant relationship was found between SNP effects on breast cancer risk and percentage density (Spearman's correlation rho = 0.0411, *P *= 0.5268). Pathway-based multi-SNP association analyses revealed no significant association between percentage density and genetic variations in the overall estrogen metabolic pathway, or any of the related subpathways (Table 3).

**Table 3:** Global genetic association tests between SNPs in the estrogen metabolic pathways and mammographic breast density

Pathway	Number of SNPs	*P *heterogeneity	*P *trend^a^
Whole pathway	239	0.840	0.507
Androgen synthesis	11	0.761	0.763
Androgen to estrogen conversion	120	0.587	0.715
Estrogen removal	134	0.834	0.872

^a^*P *values based on 5,000 permutations.

## Discussion

Our study suggests there is no appreciable effect between genetic variants involved in estrogen metabolism and mammographic density. Neither the overall estrogen metabolic pathway nor the androgen synthesis, androgen-to-estrogen conversion and estrogen removal subpathways were found to be significantly associated with mammographic density. Single SNP markers with significant associations with mammographic density were not validated in two independent datasets.

In view of estrogen exposure being a major risk factor of postmenopausal breast cancer, and mammographic density being associated with several hormone-related factors such as body mass index (increased local estrogen conversion due to increased fatty tissue), HRT, and menopausal status, the estrogen metabolic pathway has been a candidate pathway for the search of genetic variants related to mammographic density. Most of the variants in the candidate breast cancer genes evaluated in previous studies, however, have been concluded to be only weak predictors of mammographic density [10]. Association findings have been both supported and contradicted [3]. As Boyd and colleagues have discussed [34], it is likely that hormone-related factors are responsible for only a small proportion of the wide variation in mammographic density. In addition, genetic variants involved in the estrogen metabolic pathway are generally investigated based on the premise that mammographic density is an intermediate and heritable risk factor of breast cancer [4]. There is, however, accumulating evidence that mammographic density may predispose to breast cancer risk through components largely independent of estrogen metabolism [35-37].

In our study, no correlation was found between the estimates of SNP effects on breast cancer risk and mammographic density, suggesting that the same SNPs associated with breast cancer risk are not directly correlated with mammographic density. Tamimi and colleagues reported that mammographic density and circulating sex steroid levels were independently associated with breast cancer risk in postmenopausal women [35]. In addition, Kerlikowske and colleagues found no correlation between mammographic density and bone mineral density [36], both of which have been suggested to be cumulative markers of elevated estrogen exposure. Dite and colleagues performed a similar study investigating the overlap between genetic determinants of mammographic density and bone mineral density, and reported a null finding [37]. Another finding in Kerlikowske and colleagues' study was that although mammographic density remained strongly associated with elevated breast cancer risk after adjustment for hormone-related factors, the effects of bone mineral density did not [36], suggesting that estrogen metabolism plays only a small role in the effects of mammographic density on breast cancer risk.

Many studies examining the effects of exogenous estrogen exposure are in agreement with the view that estrogen has limited effects on mammographic density. Very often, the combined estrogen plus progestin regimen was found to affect mammographic density more than the estrogen-only regimen [38-41], suggesting that progestins and not estrogens are responsible for increased mammographic density. Interestingly, mammographic density is also known to have no prognostic bearing on the estrogen receptor status of breast cancer tumors [42-44], thus corroborating an estrogen/estrogen receptor independent link. Another study conducted by Vachon and colleagues found no influence of aromatase inhibitors (drugs that stop the production of estrogen in postmenopausal women) on mammographic density [45], further supporting this line of rationale.

Strengths of the present study include the large sample size and extensive coverage of SNPs in the estrogen metabolic pathway. In a review by Kelemen and colleagues, the authors summarized that previous genetic association studies exploring the relationship between the estrogen metabolic pathway and mammographic density had sample sizes ranging from between 232 and 1,260 women [3]. The number of loci involved in the estrogen metabolic pathway investigated in these studies was also limited to eight or less [3], while we examined 239 tagSNPs from 34 genes involved in the estrogen metabolic pathway. A second strength of the present study is the use of two independent populations for the validation of the associations found.

A limitation of the present work is that it includes different mammogram views across the different studies. The main study on Swedish women utilized the medio-lateral oblique view, while mammograms of the CGEMS and of the Mayo Clinic were taken using the CC view. Several studies, however, have shown correlation of densities from the medio-lateral oblique and CC views [46,47], and have shown that the different views yield similar associations with breast cancer [32]. In addition, the main focus of this study was on genetic determinants of mammographic density in postmenopausal women. Although no strong association was observed between SNPs in the estrogen metabolic pathway examined and mammographic density in postmenopausal women, whether the same lack of association between common genetic variation in the estrogen metabolism pathway and mammographic density is present in premenopausal women remains to be clarified.

## Conclusions

As mammographic density is generally considered an intermediate phenotype for breast cancer, the identification of genes that influence mammographic density would play an important role in risk prediction of breast cancer prior to the start of mammography screenings and shed light on the mechanisms behind breast cancer carcinogenesis. Overall, there is no conclusive evidence that genetic variants in genes involved in the estrogen metabolic pathway are associated with mammographic density in postmenopausal women. This knowledge will be helpful for directing the focus of future studies to alternative pathways that may be responsible for a larger bulk of the genetic component of mammographic density.

## Abbreviations

CC: craniocaudal; CGEMS: Cancer Genetic Markers of Susceptibility Project; HRT: hormone replacement therapy; SNP: single nucleotide polymorphism; tagSNP: tagging single nucleotide polymorphism.

## Competing interests

The authors declare that they have no competing interests.

## Authors' contributions

JLi participated in the study design, carried out the analyses and drafted the manuscript. LE digitized and obtained readings for the mammograms. RMT, SL, DJH, CMV, FJC and CGS contributed to the validation of this study. PL coordinated the Innovator project which contributed data on birthweight and mammographic density. JLi, KH, KC, JLiu and PH conceived of the study, and participated in its design and coordination and helped to draft the manuscript. All authors read and approved the final manuscript.

## Supplementary Material

Additional file 1 **Tables S1 to S3**. Table S1 presents a list of SNPs in the androgen synthesis subpathway and their corresponding regression coefficients and likelihood ratio test *P *values. Table S2 presents a list of SNPs in the androgen to estrogen conversion subpathway and their corresponding regression coefficients and likelihood ratio test *P *values. Table S3 presents a list of SNPs in the estrogen removal subpathway and their corresponding regression coefficients and likelihood ratio test *P *values. Click here for file

Additional file 2 **Table S4**. Table S4 presents genes containing polymorphisms within the estrogen metabolic pathway evaluated in relation to mammographic density. Click here for file

Additional file 3 **Table S5**. Table S5 presents validation results of significantly associated SNPs in the Nurses' Health Study (NHS) and the Mayo Clinic Breast Cancer Study (MBCS). Click here for file
