# Supplementary material for: Genetic variation in the estrogen metabolic pathway and mammographic density as an intermediate phenotype of breast cancer
Source: Breast Cancer Res. 2010 Mar 9;12(2):R19. doi: 10.1186/bcr2488 (PMC2879563; doi:10.1186/bcr2488)
Supplement: Additional file 1 — Tables S1 to S3. Table S1 presents a list of SNPs in the androgen synthesis subpathway and their corresponding regression coefficients and likelihood ratio test P values. Table S2 presents a list of SNPs in the androgen to estrogen conversion subpathway and their corresponding regression coefficients and likelihood ratio test P values. Table S3 presents a list of SNPs in the estrogen removal subpathway and their corresponding regression coefficients and likelihood ratio test P values. [file bcr2488-S1.DOC]

**Table S1. List of SNPs in the androgen synthesis sub-pathway and their corresponding regression coefficients and likelihood ratio test pvalues.**

| Chr | Position | SNP | Gene | N | Coefficient | SE | P |
| --- | --- | --- | --- | --- | --- | --- | --- |
| 10 | 104581383 | rs17115100 | CYP17A1 | 1665 | 0.0120 | 0.0325 | 0.7130 |
| 10 | 104584497 | rs1004467 | CYP17A1 | 1696 | -0.0148 | 0.0327 | 0.6503 |
| 10 | 104585709 | rs3781286 | CYP17A1 | 1684 | -0.0222 | 0.0206 | 0.2805 |
| 10 | 104587470 | rs2486758 | CYP17A1 | 1675 | 0.0111 | 0.0253 | 0.6601 |
| 10 | 104595318 | rs7089422 | CYP17A1 | 1692 | 0.0196 | 0.0262 | 0.4553 |
| 15 | 72403630 | rs2959008 | CYP11A1 | 1703 | 0.0475 | 0.0221 | 0.0315 |
| 15 | 72415944 | rs2959003 | CYP11A1 | 1669 | 0.0582 | 0.0224 | 0.0094 |
| 15 | 72417676 | rs2279357 | CYP11A1 | 1699 | 0.0511 | 0.0229 | 0.0260 |
| 15 | 72421952 | rs11638442 | CYP11A1 | 1677 | 0.0557 | 0.0212 | 0.0088 |
| 15 | 72449864 | rs16968478 | CYP11A1 | 1703 | 0.0575 | 0.0263 | 0.0293 |
| 15 | 72451904 | rs8039957 | CYP11A1 | 1705 | 0.0356 | 0.0303 | 0.2401 |

Chr: chromosome; SNP: single nucleotide polymorphism rsid; N: number of subjects; SE: standard error; P: P-value for 1 d.f. trend test

**Table S2. List of SNPs in the androgen to estrogen conversion sub-pathway and their corresponding regression coefficients and likelihood ratio test pvalues.**

| Chr | Position | SNP | Gene | N | Coefficient | SE | P |
| --- | --- | --- | --- | --- | --- | --- | --- |
| 1 | 119752771 | rs6428822 | HSD3B1 | 1585 | 0.0275 | 0.0209 | 0.1891 |
| 1 | 119757996 | rs4659175 | HSD3B1 | 1676 | 0.0154 | 0.0217 | 0.4775 |
| 1 | 119783549 | rs1341013 | HSD3B1 | 1692 | 0.0331 | 0.0206 | 0.1077 |
| 1 | 119800735 | rs6672903 | HSD3B1 | 1656 | -0.0163 | 0.0205 | 0.4248 |
| 1 | 119810999 | rs2298029 | HSD3B1 | 1698 | 0.0262 | 0.0219 | 0.2320 |
| 1 | 119826497 | rs911245 | HSD3B1 | 1646 | 0.0244 | 0.0218 | 0.2627 |
| 1 | 119861023 | rs10923844 | HSD3B1 | 1658 | 0.0180 | 0.0224 | 0.4221 |
| 1 | 207918699 | rs11576775 | HSD11B1 | 1644 | 0.0269 | 0.0259 | 0.2980 |
| 1 | 207925076 | rs846908 | HSD11B1 | 1694 | -0.0003 | 0.0608 | 0.9955 |
| 1 | 207933739 | rs10082248 | HSD11B1 | 1699 | -0.0040 | 0.0462 | 0.9303 |
| 1 | 207937539 | rs4844880 | HSD11B1 | 1689 | -0.0160 | 0.0254 | 0.5288 |
| 1 | 207948638 | rs2282738 | HSD11B1 | 1687 | -0.0107 | 0.0233 | 0.6442 |
| 1 | 207951994 | rs968033 | HSD11B1 | 1698 | -0.0049 | 0.0541 | 0.9283 |
| 1 | 207954341 | rs846906 | HSD11B1 | 1692 | 0.0053 | 0.0278 | 0.8487 |
| 1 | 207989777 | rs6702301 | HSD11B1 | 1687 | -0.0116 | 0.0230 | 0.6155 |
| 1 | 207996076 | rs2272866 | HSD11B1 | 1694 | 0.0521 | 0.0815 | 0.5227 |
| 2 | 31596366 | rs2208158 | SRD5A2 | 1696 | 0.0239 | 0.0220 | 0.2770 |
| 2 | 31602532 | rs3731586 | SRD5A2 | 1667 | 0.0498 | 0.0358 | 0.1635 |
| 2 | 31617062 | rs12470143 | SRD5A2 | 1627 | -0.0231 | 0.0206 | 0.2614 |
| 2 | 31620635 | rs4952197 | SRD5A2 | 1642 | 0.0322 | 0.0223 | 0.1483 |
| 2 | 31635784 | rs2268796 | SRD5A2 | 1636 | 0.0365 | 0.0208 | 0.0794 |
| 2 | 31640141 | rs2300697 | SRD5A2 | 1653 | 0.0090 | 0.0207 | 0.6627 |
| 2 | 31651315 | rs6749019 | SRD5A2 | 1616 | 0.0079 | 0.0207 | 0.7047 |
| 2 | 234175427 | rs2741019 | UGT1A1.9 | 1699 | 0.0169 | 0.0224 | 0.4521 |
| 2 | 234201376 | rs1377460 | UGT1A1.9 | 1709 | -0.0370 | 0.0254 | 0.1459 |
| 2 | 234251553 | rs7587916 | UGT1A1.9 | 1670 | -0.0142 | 0.0207 | 0.4919 |
| 2 | 234282371 | rs4663327 | UGT1A1.9 | 1691 | -0.0161 | 0.0333 | 0.6295 |
| 2 | 234295182 | rs7597496 | UGT1A1.9 | 1573 | -0.0043 | 0.0205 | 0.8321 |
| 2 | 234330521 | rs10929302 | UGT1A1.9 | 1621 | 0.0053 | 0.0226 | 0.8128 |
| 2 | 234337378 | rs6742078 | UGT1A1.9 | 1695 | 0.0047 | 0.0215 | 0.8262 |
| 2 | 234346283 | rs1042640 | UGT1A1.9 | 1709 | -0.0186 | 0.0254 | 0.4634 |
| 2 | 234348089 | rs11563250 | UGT1A1.9 | 1695 | 0.0079 | 0.0289 | 0.7831 |
| 2 | 234348502 | rs6719561 | UGT1A1.9 | 1681 | 0.0042 | 0.0215 | 0.8461 |
| 2 | 234367644 | rs10169532 | UGT1A1.9 | 1593 | -0.0147 | 0.0205 | 0.4737 |
| 2 | 234371560 | hCV256966 | UGT1A1.9 | 1677 | 0.0151 | 0.0228 | 0.5096 |
| 4 | 69904593 | rs11932983 | UGT2B11 | 1677 | 0.0203 | 0.0287 | 0.4805 |
| 4 | 69910216 | rs2331627 | UGT2B11 | 1666 | -0.0242 | 0.0262 | 0.3558 |
| 4 | 69966297 | rs10030066 | UGT2B11 | 1639 | 0.0150 | 0.0243 | 0.5363 |
| 4 | 69975780 | rs7677996 | UGT2B11 | 1602 | -0.0417 | 0.0216 | 0.0537 |
| 4 | 70024587 | rs4371687 | UGT2B11 | 1686 | -0.0110 | 0.0196 | 0.5749 |
| 4 | 70041206 | rs6837285 | UGT2B11 | 1674 | 0.0144 | 0.0196 | 0.4620 |
| 4 | 70075861 | rs6600903 | UGT2B11 | 1678 | -0.0329 | 0.0203 | 0.1053 |
| 4 | 70370761 | rs2736520 | UGT2B4 | 1660 | -0.0170 | 0.0287 | 0.5542 |
| 4 | 70370923 | rs903445 | UGT2B4 | 1663 | 0.0085 | 0.0202 | 0.6746 |
| 4 | 70375511 | rs1494798 | UGT2B4 | 1660 | 0.0173 | 0.0212 | 0.4151 |
| 4 | 70379230 | rs1080755 | UGT2B4 | 1601 | -0.0264 | 0.0239 | 0.2711 |
| 4 | 70389067 | rs2013573 | UGT2B4 | 1696 | -0.0285 | 0.0247 | 0.2483 |
| 4 | 70394283 | rs7441743 | UGT2B4 | 1528 | 0.0034 | 0.0211 | 0.8718 |
| 4 | 70397951 | rs6600771 | UGT2B4 | 1609 | 0.0456 | 0.0284 | 0.1092 |
| 5 | 6690380 | rs531241 | SRD5A1 | 1701 | 0.0202 | 0.0202 | 0.3174 |
| 5 | 6708247 | rs568509 | SRD5A1 | 1614 | 0.0047 | 0.0302 | 0.8770 |
| 5 | 6718364 | rs4702381 | SRD5A1 | 1689 | 0.0061 | 0.0239 | 0.7973 |
| 5 | 6723649 | rs16877779 | SRD5A1 | 1674 | -0.0493 | 0.0338 | 0.1454 |
| 5 | 6734187 | rs768437 | SRD5A1 | 1694 | -0.0161 | 0.0244 | 0.5107 |
| 8 | 143947798 | rs4464947 | CYP11B1 | 1696 | 0.0070 | 0.0353 | 0.8426 |
| 8 | 143952659 | rs5297 | CYP11B1 | 1701 | -0.0074 | 0.0353 | 0.8347 |
| 8 | 143989866 | rs3802230 | CYP11B2 | 1698 | 0.0163 | 0.0198 | 0.4102 |
| 8 | 143990317 | rs3097 | CYP11B2 | 1686 | 0.0064 | 0.0218 | 0.7699 |
| 8 | 143992745 | rs4543 | CYP11B2 | 1713 | 0.0058 | 0.0355 | 0.8706 |
| 8 | 143996602 | rs1799998 | CYP11B2 | 1658 | -0.0150 | 0.0196 | 0.4450 |
| 10 | 5224291 | rs1334466 | AKR1C4 | 1691 | -0.0080 | 0.0212 | 0.7060 |
| 10 | 5228196 | rs4880716 | AKR1C4 | 1684 | 0.0253 | 0.0224 | 0.2577 |
| 10 | 5233017 | rs7085249 | AKR1C4 | 1680 | 0.0203 | 0.0225 | 0.3653 |
| 10 | 5234441 | rs2151896 | AKR1C4 | 1690 | 0.0115 | 0.0199 | 0.5642 |
| 10 | 5237376 | rs3750572 | AKR1C4 | 1703 | 0.0224 | 0.0252 | 0.3752 |
| 10 | 5239111 | rs4881412 | AKR1C4 | 1698 | 0.0137 | 0.0362 | 0.7040 |
| 10 | 5240453 | rs1931679 | AKR1C4 | 1697 | 0.0384 | 0.0311 | 0.2160 |
| 10 | 5244821 | rs1831977 | AKR1C4 | 1623 | 0.0143 | 0.0264 | 0.5888 |
| 10 | 5246185 | rs12762017 | AKR1C4 | 1616 | -0.0184 | 0.0293 | 0.5313 |
| 10 | 5246497 | rs17134588 | AKR1C4 | 1646 | 0.0205 | 0.0260 | 0.4305 |
| 10 | 5248069 | rs10458795 | AKR1C4 | 1702 | 0.0181 | 0.0524 | 0.7303 |
| 15 | 49279146 | rs9972359 | CYP19A1 | 1684 | 0.0119 | 0.0203 | 0.5561 |
| 15 | 49283122 | rs934632 | CYP19A1 | 1690 | 0.0137 | 0.0253 | 0.5869 |
| 15 | 49286837 | rs7167936 | CYP19A1 | 1693 | 0.0173 | 0.0204 | 0.3957 |
| 15 | 49290136 | rs4646 | CYP19A1 | 1694 | 0.0034 | 0.0223 | 0.8775 |
| 15 | 49301213 | rs959564 | CYP19A1 | 1694 | -0.0327 | 0.0393 | 0.4059 |
| 15 | 49304392 | rs12595627 | CYP19A1 | 1657 | 0.0153 | 0.0217 | 0.4797 |
| 15 | 49324419 | hCV8234885 | CYP19A1 | 1628 | 0.0165 | 0.0208 | 0.4275 |
| 15 | 49344549 | rs12050767 | CYP19A1 | 1643 | -0.0228 | 0.0205 | 0.2667 |
| 15 | 49379835 | rs17523880 | CYP19A1 | 1697 | -0.0247 | 0.0307 | 0.4217 |
| 15 | 49382264 | hCV3060064 | CYP19A1 | 1680 | 0.0230 | 0.0205 | 0.2611 |
| 15 | 49383831 | rs8031463 | CYP19A1 | 1702 | 0.0249 | 0.0466 | 0.5927 |
| 15 | 49393870 | rs3751592 | CYP19A1 | 1670 | 0.0058 | 0.0218 | 0.7912 |
| 15 | 49397006 | rs2470150 | CYP19A1 | 1710 | -0.0271 | 0.0403 | 0.5006 |
| 15 | 49401198 | rs1902585 | CYP19A1 | 1707 | -0.0044 | 0.0205 | 0.8317 |
| 16 | 65992902 | rs11642680 | HSD11B2 | 1712 | -0.0538 | 0.0635 | 0.3968 |
| 16 | 65994359 | rs2059237 | HSD11B2 | 1702 | 0.0255 | 0.0529 | 0.6297 |
| 16 | 66007332 | rs7206718 | HSD11B2 | 1680 | 0.0237 | 0.0202 | 0.2409 |
| 16 | 66011135 | rs8047159 | HSD11B2 | 1701 | 0.0367 | 0.0323 | 0.2562 |
| 16 | 66029427 | rs4360931 | HSD11B2 | 1700 | 0.0269 | 0.0383 | 0.4831 |
| 16 | 66049072 | rs749242 | HSD11B2 | 1697 | 0.0279 | 0.0383 | 0.4660 |
| 19 | 53037973 | rs7248427 | SULT2A1 | 1675 | 0.0073 | 0.0205 | 0.7207 |
| 19 | 53040066 | rs17239147 | SULT2A1 | 1686 | -0.0033 | 0.0289 | 0.9084 |
| 19 | 53048964 | rs4483956 | SULT2A1 | 1688 | 0.0282 | 0.0207 | 0.1720 |
| 19 | 53063945 | rs188440 | SULT2A1 | 1706 | 0.0047 | 0.0228 | 0.8359 |
| 19 | 53067510 | rs296364 | SULT2A1 | 1678 | -0.0034 | 0.0196 | 0.8627 |
| 19 | 53074328 | rs11083905 | SULT2A1 | 1706 | -0.0181 | 0.0336 | 0.5897 |
| 19 | 53083388 | rs7508610 | SULT2A1 | 1642 | 0.0005 | 0.0210 | 0.9797 |
| 19 | 53090448 | rs2972612 | SULT2A1 | 1648 | 0.0002 | 0.0227 | 0.9942 |
| 19 | 53745118 | rs279451 | SULT2B1 | 1659 | -0.0005 | 0.0287 | 0.9875 |
| 19 | 53747608 | rs279447 | SULT2B1 | 1715 | 0.0142 | 0.0482 | 0.7680 |
| 19 | 53753536 | rs3848542 | SULT2B1 | 1694 | -0.0318 | 0.0227 | 0.1613 |
| 19 | 53756771 | rs12611137 | SULT2B1 | 1700 | 0.0005 | 0.0259 | 0.9858 |
| 19 | 53762686 | rs2665605 | SULT2B1 | 1703 | -0.0128 | 0.0300 | 0.6703 |
| 19 | 53766894 | rs2665577 | SULT2B1 | 1701 | 0.0057 | 0.0212 | 0.7865 |
| 19 | 53775305 | rs6509396 | SULT2B1 | 1700 | -0.0125 | 0.0210 | 0.5525 |
| 19 | 53784242 | rs10426628 | SULT2B1 | 1679 | 0.0335 | 0.0234 | 0.1522 |
| 19 | 53791303 | rs2665587 | SULT2B1 | 1706 | 0.0085 | 0.0282 | 0.7629 |
| 19 | 53791767 | rs3815691 | SULT2B1 | 1706 | 0.0382 | 0.0298 | 0.2003 |
| 19 | 53794211 | rs1132054 | SULT2B1 | 1679 | 0.0071 | 0.0203 | 0.7282 |
| 19 | 53812246 | rs369880 | SULT2B1 | 1691 | -0.0108 | 0.0261 | 0.6799 |
| 23 | 7125093 | rs707762 | STS | 1687 | 0.0435 | 0.0205 | 0.0340 |
| 23 | 7180925 | rs2270112 | STS | 1686 | -0.0485 | 0.0208 | 0.0197 |
| 23 | 7184199 | rs12861247 | STS | 1701 | 0.0081 | 0.0328 | 0.8050 |
| 23 | 7194615 | rs5934850 | STS | 1617 | 0.0373 | 0.0209 | 0.0744 |
| 23 | 7224805 | rs5934914 | STS | 1587 | -0.0008 | 0.0231 | 0.9722 |
| 23 | 7246970 | rs17268974 | STS | 1605 | 0.0503 | 0.0238 | 0.0349 |
| 23 | 7253304 | rs4403552 | STS | 1694 | 0.0072 | 0.0245 | 0.7683 |
| 23 | 7264481 | rs17268988 | STS | 1687 | -0.0270 | 0.0228 | 0.2362 |
| 23 | 7280996 | rs1131289 | STS | 1691 | 0.0079 | 0.0220 | 0.7195 |

Chr: chromosome; SNP: single nucleotide polymorphism rsid; N: number of subjects; SE: standard error; P: P-value for 1 d.f. trend test

**Table S3. List of SNPs in estrogen removal sub-pathway and their corresponding regression coefficients and likelihood ratio test pvalues.**

| Chr | Position | SNP | Gene | N | Coefficient | SE | P |
| --- | --- | --- | --- | --- | --- | --- | --- |
| 1 | 159531389 | hCV2765051 | HSD17B7 | 1662 | -0.0150 | 0.0302 | 0.6188 |
| 1 | 161014649 | rs1780007 | HSD17B7 | 1700 | -0.0230 | 0.0252 | 0.3602 |
| 1 | 161043150 | rs1039874 | HSD17B7 | 1686 | -0.0205 | 0.0485 | 0.6719 |
| 1 | 161061083 | rs1704767 | HSD17B7 | 1676 | 0.0097 | 0.0203 | 0.6338 |
| 1 | 161061423 | rs1006390 | HSD17B7 | 1670 | -0.0115 | 0.0237 | 0.6273 |
| 2 | 38136239 | rs163076 | CYP1B1 | 1672 | -0.0295 | 0.0212 | 0.1634 |
| 2 | 38145208 | rs2256327 | CYP1B1 | 1659 | 0.0250 | 0.0250 | 0.3166 |
| 2 | 38146266 | rs163086 | CYP1B1 | 1677 | 0.0125 | 0.0248 | 0.6150 |
| 2 | 38151707 | rs1056836 | CYP1B1 | 1682 | 0.0047 | 0.0200 | 0.8154 |
| 2 | 38156298 | rs2551188 | CYP1B1 | 1693 | -0.0046 | 0.0218 | 0.8337 |
| 2 | 234175427 | rs2741019 | UGT1A1.9 | 1699 | 0.0169 | 0.0224 | 0.4521 |
| 2 | 234201376 | rs1377460 | UGT1A1.9 | 1709 | -0.0370 | 0.0254 | 0.1459 |
| 2 | 234251553 | rs7587916 | UGT1A1.9 | 1670 | -0.0142 | 0.0207 | 0.4919 |
| 2 | 234282371 | rs4663327 | UGT1A1.9 | 1691 | -0.0161 | 0.0333 | 0.6295 |
| 2 | 234295182 | rs7597496 | UGT1A1.9 | 1573 | -0.0043 | 0.0205 | 0.8321 |
| 2 | 234330521 | rs10929302 | UGT1A1.9 | 1621 | 0.0053 | 0.0226 | 0.8128 |
| 2 | 234337378 | rs6742078 | UGT1A1.9 | 1695 | 0.0047 | 0.0215 | 0.8262 |
| 2 | 234346283 | rs1042640 | UGT1A1.9 | 1709 | -0.0186 | 0.0254 | 0.4634 |
| 2 | 234348089 | rs11563250 | UGT1A1.9 | 1695 | 0.0079 | 0.0289 | 0.7831 |
| 2 | 234348502 | rs6719561 | UGT1A1.9 | 1681 | 0.0042 | 0.0215 | 0.8461 |
| 2 | 234367644 | rs10169532 | UGT1A1.9 | 1593 | -0.0147 | 0.0205 | 0.4737 |
| 2 | 234371560 | hCV256966 | UGT1A1.9 | 1677 | 0.0151 | 0.0228 | 0.5096 |
| 4 | 69904593 | rs11932983 | UGT2B11 | 1677 | 0.0203 | 0.0287 | 0.4805 |
| 4 | 69910216 | rs2331627 | UGT2B11 | 1666 | -0.0242 | 0.0262 | 0.3558 |
| 4 | 69966297 | rs10030066 | UGT2B11 | 1639 | 0.0150 | 0.0243 | 0.5363 |
| 4 | 69975780 | rs7677996 | UGT2B11 | 1602 | -0.0417 | 0.0216 | 0.0537 |
| 4 | 70024587 | rs4371687 | UGT2B11 | 1686 | -0.0110 | 0.0196 | 0.5749 |
| 4 | 70041206 | rs6837285 | UGT2B11 | 1674 | 0.0144 | 0.0196 | 0.4620 |
| 4 | 70075861 | rs6600903 | UGT2B11 | 1678 | -0.0329 | 0.0203 | 0.1053 |
| 4 | 70370761 | rs2736520 | UGT2B4 | 1660 | -0.0170 | 0.0287 | 0.5542 |
| 4 | 70370923 | rs903445 | UGT2B4 | 1663 | 0.0085 | 0.0202 | 0.6746 |
| 4 | 70375511 | rs1494798 | UGT2B4 | 1660 | 0.0173 | 0.0212 | 0.4151 |
| 4 | 70379230 | rs1080755 | UGT2B4 | 1601 | -0.0264 | 0.0239 | 0.2711 |
| 4 | 70389067 | rs2013573 | UGT2B4 | 1696 | -0.0285 | 0.0247 | 0.2483 |
| 4 | 70394283 | rs7441743 | UGT2B4 | 1528 | 0.0034 | 0.0211 | 0.8718 |
| 4 | 70397951 | rs6600771 | UGT2B4 | 1609 | 0.0456 | 0.0284 | 0.1092 |
| 4 | 70736595 | rs1529039 | STE..SULT1E1. | 1685 | -0.0463 | 0.0297 | 0.1197 |
| 4 | 70740955 | rs1220725 | STE..SULT1E1. | 1522 | 0.0032 | 0.0321 | 0.9212 |
| 4 | 70743796 | rs3775779 | STE..SULT1E1. | 1659 | 0.0274 | 0.0216 | 0.2055 |
| 4 | 70752594 | rs4149534 | STE..SULT1E1. | 1690 | -0.0096 | 0.0227 | 0.6732 |
| 4 | 70753566 | rs1220716 | STE..SULT1E1. | 1691 | -0.0843 | 0.0483 | 0.0812 |
| 4 | 70760988 | rs4149525 | STE..SULT1E1. | 1672 | 0.0165 | 0.0278 | 0.5513 |
| 4 | 70774109 | rs1154741 | STE..SULT1E1. | 1700 | -0.0059 | 0.0214 | 0.7830 |
| 5 | 118797197 | rs154632 | HSD17B4 | 1668 | 0.0107 | 0.0220 | 0.6273 |
| 5 | 118799322 | rs13154090 | HSD17B4 | 1703 | -0.0328 | 0.0489 | 0.5023 |
| 5 | 118816919 | rs10478424 | HSD17B4 | 1690 | -0.0072 | 0.0232 | 0.7550 |
| 5 | 118820620 | rs11749784 | HSD17B4 | 1694 | -0.0236 | 0.0239 | 0.3247 |
| 5 | 118830120 | rs1283826 | HSD17B4 | 1697 | -0.0409 | 0.0382 | 0.2836 |
| 5 | 118835035 | rs439954 | HSD17B4 | 1602 | 0.0468 | 0.0303 | 0.1224 |
| 5 | 118860864 | rs3756513 | HSD17B4 | 1688 | -0.0055 | 0.0315 | 0.8607 |
| 5 | 118904980 | rs17388769 | HSD17B4 | 1695 | -0.0169 | 0.0303 | 0.5782 |
| 6 | 33266876 | rs2269346 | HSD17B8 | 1669 | 0.0160 | 0.0471 | 0.7343 |
| 6 | 33270060 | rs2072915 | HSD17B8 | 1676 | -0.0132 | 0.0222 | 0.5530 |
| 6 | 33277873 | rs1547387 | HSD17B8 | 1708 | 0.0062 | 0.0343 | 0.8567 |
| 6 | 33280910 | rs110662 | HSD17B8 | 1608 | -0.0046 | 0.0219 | 0.8321 |
| 6 | 160018212 | rs4342445 | SOD2 | 1691 | 0.0393 | 0.0238 | 0.0985 |
| 6 | 160020106 | rs2842980 | SOD2 | 1685 | -0.0171 | 0.0251 | 0.4968 |
| 6 | 160023074 | rs5746136 | SOD2 | 1676 | 0.0185 | 0.0220 | 0.3996 |
| 6 | 160027081 | rs1800665 | SOD2 | 1684 | -0.0548 | 0.0956 | 0.5664 |
| 6 | 160030444 | rs2758334 | SOD2 | 1645 | -0.0032 | 0.0200 | 0.8712 |
| 7 | 99013350 | hCV11246907 | CYP3A4_5 | 1705 | 0.0061 | 0.0586 | 0.9174 |
| 7 | 99083016 | rs4646457 | CYP3A4_5 | 1680 | 0.0184 | 0.0386 | 0.6339 |
| 7 | 99104254 | rs4646450 | CYP3A4_5 | 1670 | 0.0143 | 0.0285 | 0.6170 |
| 7 | 99142648 | rs2687078 | CYP3A4_5 | 1691 | 0.0077 | 0.0349 | 0.8254 |
| 7 | 99170019 | rs2687133 | CYP3A4_5 | 1706 | -0.0009 | 0.0379 | 0.9816 |
| 7 | 99186264 | rs6945984 | CYP3A4_5 | 1622 | -0.0127 | 0.0321 | 0.6923 |
| 8 | 18110372 | rs11203942 | NAT1 | 1673 | -0.0115 | 0.0210 | 0.5860 |
| 8 | 18113444 | rs3850751 | NAT1 | 1711 | -0.0152 | 0.0202 | 0.4508 |
| 8 | 18118222 | rs6586714 | NAT1 | 1689 | 0.0508 | 0.0321 | 0.1135 |
| 8 | 18120186 | rs4921880 | NAT1 | 1692 | 0.0012 | 0.0238 | 0.9592 |
| 8 | 18120277 | rs11777998 | NAT1 | 1694 | -0.0150 | 0.0355 | 0.6715 |
| 8 | 18121590 | rs7003890 | NAT1 | 1691 | -0.0100 | 0.0202 | 0.6218 |
| 8 | 18122267 | rs8190837 | NAT1 | 1700 | -0.0269 | 0.0330 | 0.4159 |
| 8 | 18287058 | rs4921906 | NAT2 | 1685 | 0.0268 | 0.0202 | 0.1860 |
| 8 | 18295202 | rs9987109 | NAT2 | 1703 | 0.0232 | 0.0204 | 0.2550 |
| 8 | 18298747 | rs2410556 | NAT2 | 1594 | -0.0012 | 0.0319 | 0.9693 |
| 8 | 18306826 | rs4646257 | NAT2 | 1688 | -0.0243 | 0.0257 | 0.3443 |
| 8 | 18307392 | rs1495748 | NAT2 | 1680 | -0.0077 | 0.0216 | 0.7220 |
| 8 | 18309403 | rs1495738 | NAT2 | 1673 | 0.0355 | 0.0207 | 0.0860 |
| 8 | 18316718 | rs4921914 | NAT2 | 1693 | -0.0011 | 0.0246 | 0.9630 |
| 9 | 98026168 | rs442686 | HSD17B3 | 1662 | -0.0421 | 0.0216 | 0.0513 |
| 9 | 98027222 | rs4306016 | HSD17B3 | 1623 | 0.0067 | 0.0202 | 0.7389 |
| 9 | 98043085 | rs2066485 | HSD17B3 | 1703 | 0.0668 | 0.0293 | 0.0230 |
| 9 | 98058102 | rs8190534 | HSD17B3 | 1685 | 0.0359 | 0.0247 | 0.1460 |
| 9 | 98061403 | rs7039978 | HSD17B3 | 1694 | -0.0632 | 0.0203 | 0.0019 |
| 9 | 98069778 | rs8190530 | HSD17B3 | 1708 | 0.0105 | 0.0203 | 0.6062 |
| 9 | 98091939 | rs7022250 | HSD17B3 | 1696 | -0.0351 | 0.0208 | 0.0915 |
| 9 | 98104670 | rs8190479 | HSD17B3 | 1619 | -0.0058 | 0.0396 | 0.8845 |
| 11 | 67100533 | rs656652 | GSTP1 | 1705 | -0.0108 | 0.0201 | 0.5921 |
| 15 | 72790561 | rs6495121 | CYP1A1.2 | 1680 | 0.0390 | 0.0304 | 0.2008 |
| 15 | 72800040 | rs1799814 | CYP1A1.2 | 1698 | -0.0215 | 0.0563 | 0.7026 |
| 15 | 72806502 | rs2470893 | CYP1A1.2 | 1702 | 0.0161 | 0.0213 | 0.4493 |
| 15 | 72814933 | rs2472297 | CYP1A1.2 | 1665 | 0.0120 | 0.0223 | 0.5910 |
| 15 | 72839115 | rs1350194 | CYP1A1.2 | 1711 | -0.0556 | 0.0625 | 0.3737 |
| 16 | 28507783 | rs17639997 | SULT1A1.2 | 1714 | -0.0244 | 0.0386 | 0.5280 |
| 16 | 28517197 | rs12445705 | SULT1A1.2 | 1562 | -0.0116 | 0.0455 | 0.7994 |
| 16 | 28521466 | rs11074907 | SULT1A1.2 | 1609 | 0.0273 | 0.0204 | 0.1814 |
| 16 | 28523209 | rs11074904 | SULT1A1.2 | 1697 | -0.0230 | 0.0318 | 0.4701 |
| 16 | 28524629 | rs6839 | SULT1A1.2 | 1569 | 0.0168 | 0.0205 | 0.4147 |
| 16 | 28539522 | rs2411453 | SULT1A1.2 | 1618 | 0.0345 | 0.0210 | 0.1004 |
| 16 | 68287295 | rs12595869 | NQO1 | 1698 | -0.0493 | 0.0269 | 0.0672 |
| 16 | 68287927 | rs1437134 | NQO1 | 1637 | 0.0184 | 0.0201 | 0.3614 |
| 16 | 68288056 | rs3826154 | NQO1 | 1653 | -0.0056 | 0.0288 | 0.8449 |
| 16 | 68299549 | rs12933210 | NQO1 | 1669 | -0.0314 | 0.0206 | 0.1279 |
| 16 | 68321913 | rs1469908 | NQO1 | 1695 | -0.0472 | 0.0206 | 0.0223 |
| 16 | 68329211 | hCV26055094 | NQO1 | 1677 | 0.0310 | 0.0200 | 0.1220 |
| 16 | 68333878 | rs1075935 | NQO1 | 1658 | -0.0377 | 0.0527 | 0.4754 |
| 16 | 80622339 | rs4291899 | HSD17B2 | 1707 | -0.0508 | 0.0349 | 0.1457 |
| 16 | 80632818 | rs11648233 | HSD17B2 | 1694 | 0.0043 | 0.0209 | 0.8361 |
| 16 | 80654301 | rs11642323 | HSD17B2 | 1700 | 0.0131 | 0.0211 | 0.5359 |
| 16 | 80670972 | rs2042429 | HSD17B2 | 1624 | 0.0083 | 0.0207 | 0.6889 |
| 16 | 80672242 | rs2966244 | HSD17B2 | 1710 | 0.0853 | 0.0711 | 0.2308 |
| 16 | 80683051 | rs1017243 | HSD17B2 | 1686 | -0.0063 | 0.0208 | 0.7635 |
| 16 | 80690493 | rs996752 | HSD17B2 | 1620 | -0.0153 | 0.0212 | 0.4700 |
| 16 | 80693012 | rs10514525 | HSD17B2 | 1680 | 0.0168 | 0.0204 | 0.4105 |
| 16 | 80693755 | rs1364284 | HSD17B2 | 1686 | 0.0020 | 0.0312 | 0.9497 |
| 16 | 80700383 | rs7200459 | HSD17B2 | 1699 | -0.0013 | 0.0373 | 0.9724 |
| 16 | 80703426 | rs12597465 | HSD17B2 | 1693 | 0.0152 | 0.0205 | 0.4605 |
| 17 | 37958089 | rs2830 | HSD17B1 | 1578 | -0.0031 | 0.0214 | 0.8841 |
| 17 | 37964418 | rs2854977 | HSD17B1 | 1688 | 0.0360 | 0.0434 | 0.4076 |
| 17 | 37974568 | rs650558 | HSD17B1 | 1700 | -0.0176 | 0.0231 | 0.4452 |
| 17 | 37975688 | rs1474040 | HSD17B1 | 1682 | 0.0039 | 0.0252 | 0.8763 |
| 17 | 37981755 | rs878291 | HSD17B1 | 1689 | -0.0073 | 0.0205 | 0.7230 |
| 17 | 37988603 | rs9903251 | HSD17B1 | 1691 | 0.0275 | 0.0218 | 0.2070 |
| 22 | 18291831 | rs12484658 | COMT | 1701 | 0.0322 | 0.0396 | 0.4167 |
| 22 | 18314051 | rs174675 | COMT | 1674 | -0.0065 | 0.0229 | 0.7766 |
| 22 | 18317638 | rs5993883 | COMT | 1689 | -0.0046 | 0.0202 | 0.8199 |
| 22 | 18329644 | rs3810595 | COMT | 1664 | 0.0115 | 0.0209 | 0.5829 |
| 22 | 18331897 | rs4646315 | COMT | 1701 | 0.0113 | 0.0239 | 0.6348 |
| 22 | 18332561 | rs165774 | COMT | 1694 | -0.0032 | 0.0220 | 0.8861 |
| 22 | 18333176 | rs174696 | COMT | 1695 | -0.0128 | 0.0241 | 0.5952 |
| 22 | 18335157 | rs9306235 | COMT | 1703 | -0.0084 | 0.0408 | 0.8364 |
| 22 | 18349075 | rs2073747 | COMT | 1675 | 0.0015 | 0.0245 | 0.9525 |
| 22 | 18350502 | rs1990277 | COMT | 1692 | 0.0017 | 0.0207 | 0.9327 |

Chr: chromosome; SNP: single nucleotide polymorphism rsid; N: number of subjects; SE: standard error; P: P-value for 1 d.f. trend test
