# Supplementary material for: Genetic variation in the estrogen metabolic pathway and mammographic density as an intermediate phenotype of breast cancer
Source: Breast Cancer Res. 2010 Mar 9;12(2):R19. doi: 10.1186/bcr2488 (PMC2879563; doi:10.1186/bcr2488)
Supplement: Additional file 2 — Table S4. Table S4 presents genes containing polymorphisms within the estrogen metabolic pathway evaluated in relation to mammographic density. [file bcr2488-S2.DOC]

**Table S4. Genes containing polymorphisms within the estrogen metabolic pathway evaluated in relation to mammographic density.**

| *Gene* | *Full name* | *Locus* | *No. of SNPs* | *Reference* |
| --- | --- | --- | --- | --- |
| AKR1C4 | Aldo-keto reductase family 1, member C4 | 10p15-14 | 11 | [12] |
| COMT | catechol-o-methyltransferase | 22q11.2 | 10 | [5, 10, 11, 14, 48] |
| CYP11A1 | cytochrome p450scc / cholesterol side chain cleavage | 15q23-24 | 6 |  |
| CYP11B1 | cytochrome p450 / steroid 11 beta 1 hydroxylase | 8q21 | 2 |  |
| CYP11B2 | cytochrome p450 / steroid 11 beta 2 hydroxylase | 8q21 | 4 |  |
| CYP17A1 | cytochrome p450 / 17 alfa steroid hydroxylase | 10q24.3 | 5 | [10, 11, 13, 14] |
| CYP19A1 | cytochrome p450 / aromatase | 15q21.1 | 14 | [10, 49] |
| CYP1A1-2 | cytochrome p450 | 15q22-qter | 5 | [10, 11, 48] |
| CYP1B1 | cytochrome p450 | 2p22-21 | 5 | [10-12] |
| CYP3A4_5 | cytochrome p450 | 7q22.1 | 6 |  |
| GSTP1 | Glutathione S transferase, pi | 11q13 | 1 |  |
| HSD11B1 | 11 beta hydroxy steroid dehydrogenase 1 | 1q32-q41 | 9 |  |
| HSD11B2 | 11 beta hydroxy steroid dehydrogenase 2 | 16q22 | 6 |  |
| HSD17B1 | 17 beta hydroxy steroid dehydrogenase 1 | 17q12-21 | 6 | [14] |
| HSD17B2 | 17 beta hydroxy steroid dehydrogenase 2 | 16q24.1-24.2 | 11 |  |
| HSD17B3 | 17 beta hydroxy steroid dehydrogenase 3 | 9q22 | 8 |  |
| HSD17B4 | 17 beta hydroxy steroid dehydrogenase 4 | 5q2 | 8 |  |
| HSD17B7 | 17 beta hydroxy steroid dehydrogenase 7 | 10p11.2 | 5 |  |
| HSD17B8 | 17 beta hydroxy steroid dehydrogenase 8 | 6p21.3 | 4 |  |
| HSD3B1 | 3 beta hydroxy steroid dehydrogenase 1 | 1p13.1 | 7 | [5, 14] |
| NAT1 | N-acetyltransferase 1 | 8p23.1-21.3 | 7 |  |
| NAT2 | N-acetyltransferase 2 | 8p23.1-21.3 | 7 |  |
| NQO1 | NAD(P)H dehydrogenase, quinone 1 | 16q22.1 | 7 |  |
| SOD2 | Superoxide dismutase 2, mitochondrial | 6q25.3 | 5 |  |
| SRD5A1 | steroid 5 alpha reductase 1 | 5p15 | 5 |  |
| SRD5A2 | steroid 5 alpha reductase 2 | 2p23 | 7 |  |
| STE (SULT1E1) | estrogen/aryl sulfotransferase | 4q13.1 | 7 |  |
| STS | arylsulfatase C/steroid sulfatase | Xp22.32 | 9 |  |
| SULT1A1-2 | sulfotransferase family 1A, phenol-preferring member 1 | 16p12.1-11.2 | 6 |  |
| SULT2A1 | dehydroepiandrosterone sulfotransferase | 19q13.3 | 8 |  |
| SULT2B1 | sulfotransferase family 2B member 1 | 19q13.3 | 12 |  |
| UGT1A1-9 | Uridine diphosphate glucuronosyltransferase 1 family, polypeptide A9 | 2q37 | 12 | [10] |
| UGT2B11 | Uridine diphosphate glucuronosyltransferase 2 family, member B 11 | 4q13.2 | 7 |  |
| UGT2B4 | Uridine diphosphate glucuronosyltransferase 2 family, member B 4 | 4q13 | 7 | [12] |
