# Supplementary material for: Genetic variation in the estrogen metabolic pathway and mammographic density as an intermediate phenotype of breast cancer
Source: Breast Cancer Res. 2010 Mar 9;12(2):R19. doi: 10.1186/bcr2488 (PMC2879563; doi:10.1186/bcr2488)
Supplement: Additional file 3 — Table S5. Table S5 presents validation results of significantly associated SNPs in the Nurses' Health Study (NHS) and the Mayo Clinic Breast Cancer Study (MBCS). [file bcr2488-S3.DOC]

**Table S5. Validation results of significantly associated SNPs in the Nurses’ Health Study (NHS) and the Mayo Clinic Breast Cancer Study (MBCS)**

| SNP | Gene | Study | Minor allele | MAF | N | Genotyped* | R2 | Coefficient | SE | P |
| --- | --- | --- | --- | --- | --- | --- | --- | --- | --- | --- |
| rs11638442 | CYP11A1 | NHS | C | 0.40 | 1590 | No | 1.00 | -0.030 | 0.020 | 0.12 |
| rs11638442 | CYP11A1 | MBCS | C | 0.40 | 783 | Yes | 1.00 | -0.005 | 0.034 | 0.88 |
| rs16968478 | CYP11A1 | NHS | G | 0.19 | 1590 | Yes | 1.00 | -0.036 | 0.026 | 0.16 |
| rs2279357 | CYP11A1 | NHS | T | 0.29 | 1590 | Yes | 1.00 | -0.0002 | 0.022 | 0.99 |
| rs2959003 | CYP11A1 | NHS | A | 0.33 | 1590 | No | 1.00 | -0.020 | 0.021 | 0.35 |
| rs2959008 | CYP11A1 | NHS | A | 0.33 | 1590 | No | 1.00 | -0.021 | 0.021 | 0.32 |
|  |  |  |  |  |  |  |  |  |  |  |
| rs2066485 | HSD17B3 | NHS | C | 0.15 | 1590 | No | 1.00 | -0.018 | 0.028 | 0.52 |
| rs7039978 | HSD17B3 | NHS | A | 0.48 | 1590 | No | 0.97 | 0.029 | 0.020 | 0.15 |
|  |  |  |  |  |  |  |  |  |  |  |
| rs1469908 | NQO1 | NHS | C | 0.43 | 1590 | No | 0.99 | -0.002 | 0.020 | 0.94 |
|  |  |  |  |  |  |  |  |  |  |  |
| rs17268974 | STS | NHS | A | 0.22 | 1590 | No | 0.88 | 0.0003 | 0.026 | 0.99 |
| rs2270112 | STS | NHS | C | 0.32 | 1590 | No | 1.00 | 0.020 | 0.021 | 0.35 |
| rs707762 | STS | NHS | A | 0.40 | 1590 | No | 1.00 | 0.021 | 0.020 | 0.31 |
|  |  |  |  |  |  |  |  |  |  |  |

* SNPs not genotyped were imputed using MACH based on HapMap Phase II (release 21a).

SNP: single nucleotide polymorphism rsid; NHS: Nurses’ Healthy study; MBCS: The Mayo Clinic Breast Cancer Study; MAF: minor allele frequency; N: number of subjects; SE: standard error; P: P-value for 1 d.f. trend test
